# Effect of *Angelica sinensis* Polysaccharides on Osteoarthritis *In Vivo* and *In Vitro*: A Possible Mechanism to Promote Proteoglycans Synthesis

**DOI:** 10.1155/2013/794761

**Published:** 2013-06-04

**Authors:** Jun Qin, Yan-song Liu, Jun Liu, Jing Li, Yang Tan, Xiao-jun Li, Jacques Magdalou, Qi-bing Mei, Hui Wang, Liao-bin Chen

**Affiliations:** ^1^Department of Orthopaedic Surgery, Zhongnan Hospital, Wuhan University, Wuhan, Hubei 430071, China; ^2^Department of Pharmacology, Basic Medical School, Wuhan University, Wuhan, Hubei 430071, China; ^3^UMR7561 CNRS-UHP, Laboratoire de Physiopathologie et Pharmacologie Articulaires, Faculté de Médecine, Vandoeuvre-lès-Nancy Cedex, 54505 Lorraine, France; ^4^Department of Pharmacology, School of Pharmacy, The Fourth Military Medical University, Xi'an, Shanxi 710032, China

## Abstract

This study investigated the effect of *Angelica sinensis* polysaccharides (APS-3c) on rat osteoarthritis (OA) model *in vivo* and rat interleukin-1-beta- (IL-1**β**-) stimulated chondrocytes *in vitro*. APS-3c was administrated into rat OA knee joints and had protective effects on rat OA cartilage *in vivo*. Primary rat articular chondrocytes were cotreated with APS-3c and IL-1**β** *in vitro*. 2~50 **μ**g/mL APS-3c had no effect on chondrocytes viability, whereas it increased the proteoglycans (PGs) synthesis inhibited by IL-1**β**. Microarray analysis showed that the significant changes were concentrated in the genes which were involved in PGs synthesis. RT-PCR confirmed that treatment with APS-3c increased the mRNA expression of aggrecan and glycosyltransferases (GTs) inhibited by IL-1**β** but did not affect the mRNA expression of matrix-degrading enzymes. These results indicate that APS-3c can improve PGs synthesis of chondrocytes on rat OA model *in vivo* and IL-1**β**-stimulated chondrocytes *in vitro*, which is due to the promotion of the expression of aggrecan and GTs involved in PGs synthesis but not the inhibition of the expression of matrix-degrading enzymes. Our findings suggest the clinical relevance of APS-3c in the prospective of future alternative medical treatment for OA.

## 1. Introduction

Osteoarthritis (OA) is the most common clinical syndrome of joint pain accompanied by varying degrees of functional limitation and reduced quality of life [1]. OA is characterized by loss of articular cartilage components, mainly proteoglycans (PGs), leading to tissue destruction and hypocellularity, eventually resulting in loss of joint function [2]. The incidence is increasing with the lengthening of life expectancy, creating a major public health challenge for the coming years [3]. Current pharmacological treatments focus on reduction of pain and increase mobility to improve overall quality of life, but their efficacy on OA progression is limited. Glucosamine and chondroitin are only used for complementary medicines and their clinical effectiveness causes many controversies [4]. For these reasons, new treatment agents for OA are needed.

A hallmark of OA is the marked increase in proinflammatory cytokines, such as interleukin 1 beta (IL-1*β*), that inhibit the synthesis of PGs and collagen and enhance their degradation [5]. Stimulation of PGs synthesis and/or inhibition of PGs degradation, therefore, are of central importance for OA treatment. Aggrecan, the large aggregating PG in the extracellular matrix (ECM) of articular cartilage, maintains hydration, withstands compression, and interacts with other macromolecules. It comprises chondroitin sulfate (CS) and keratan sulfate (KS) glycosaminoglycan (GAG) chains covalently linked to a core protein [6]. CS is a main component of the cartilage matrix and consists of repeating disaccharide units of N-acetylgalactosamine and glucuronic acid residues [6]. Aggrecanase-1 and -2 have been found to be responsible for aggrecan cleavage [7, 8]. Metalloproteinase 3 (MMP-3) not only cleaves aggrecan, but also activates the proforms of several MMPs and contributes to the activation of aggrecanase-2 [9, 10]. The biosynthesis of GAG chains is initiated by the formation of a tetrasaccharide, GlcA*β*1, 3Gal*β*1, 3Gal*β*1, and 4Xyl*β*-O attached to the hydroxyl group of specific serine residues. This linkage tetrasaccharide is formed by the stepwise addition of each sugar residue catalyzed by glycosyltransferases (GTs) [11]. IL-1*β* can downregulate the gene expression and activity of GTs and then reduces the biosynthesis of GAGs in chondrocytes [12, 13]. IL-1*β* can also inhibit chondrocytes proliferation and promote chondrocytes apoptosis [14].

*Angelica sinensis* (Oliv.) Diels (Apiaceae), the root of which is known in Chinese as Danggui, is first documented in Shennong Bencao Jing (Shennong's Materia Medica; 200–300 AD) and used as an ingredient to treat arthritis and rheumatism, modulate the immune system, prevent platelet aggregation, and act as an anti-inflammatory in Chinese traditional therapy [15, 16]. Over 70 compounds have been identified from *Angelica sinensis*, including essential oils, ferulic acid (FA), *Angelica sinensis* polysaccharides (APS), phthalide dimers, polyacetylenes, vitamins, and amino acids [17]. Recently, for the first time our laboratory showed the beneficial effect of FA on OA [18]. We showed that FA prevents chondrocytes apoptosis and attenuates the levels of metalloproteinase (MMPs)/tissue inhibitor of metalloproteinase (TIMPs) to prevent PG degradation in human OA chondrocytes [18]. To elucidate the whole mechanisms of *Angelica sinensis*, researchers have recently been interested in the gastrointestinal protective effects, immunomodulatory [19] and antitumor activity of APS [20, 21]. APS, which is extracted with water as the initial extraction solvent, consists of xylose, galactose, glucose, arabinose, rhamnose, fucose, and glucuronic acid [22, 23]. The biosynthesis of GAG chains involves the ordered stepwise addition of a series of monosaccharide mentioned above through the action of specific GTs. 

The aim of this study is to investigate the possibility that APS-3c promotes PGs synthesis both *in vitro* in cultured rat IL-1*β*-stimulated chondrocytes and *in vivo* in OA animal model. To get insight the possible mechanism of APS-3c to promote PGs synthesis in this study, we explored the hypothesis that APS-3c may promote the gene and protein expression of GTs. To confirm the precise molecular mechanisms of APS-3c on OA, we also investigated cell proliferation, cell apoptosis, and the gene expression of matrix-degrading enzymes in rat IL-1*β*-stimulated chondrocytes. Until now, there has been no study on the effect of APS-3c on OA. This study might be helpful in developing a new treatment for OA.

## 2. Materials and Methods

### 2.1. Preparation of *Angelica sinensis* Polysaccharides

The roots of *A. sinensis* were collected in Minxian County, Gansu Province, China. The coarse powder of the roots was air dried in the shade and stored in a well-closed vessel for use. APS-3c was prepared as described previously [24] and presented by Dr. Mei Qi-bing, from the Fourth Military Medical University. APS-3c appeared as white powder. The HPSEC profiles demonstrated that APS-3c was eluted as single and symmetrical peaks, which indicated that it was homogeneous polysaccharides. The molecular weight of APS-3c was determined to be 1.4 × 10^4^ Da by high-performance gel-permeation chromatography. The percentage of total sugar was determined to be 61.0% by phenol sulfuric acid method. The component sugars of APS-3c, determined by GC, were glucose, galactose, arabinose, rhamnose, and mannose and xylose in a molar ratio of 6.3 : 4.7 : 6.7 : 6.5 : 1.6 : 1.0. The limulus amebocytes lysate (LAL) result showed that APS-3c was free of LPS contamination.

### 2.2. *In Vivo* Experiments

Animal care and treatment were in accordance with the Guidelines of the Laboratory Animal Management and Review Committee of Wuhan University (China). Forty-eight male Wistar rats (8 weeks old, Animal Center of Wuhan University) were separated into six groups of eight rats each: (i) normal control group; (ii) APS-3c control group: normal rats treated with 0.05% APS-3c; (iii) OA model control group: OA rats induced by 4% papain; (iv) APS-3c treatment groups, OA rats treated with 0.01%, 0.025%, and 0.05% APS-3c, respectively. The OA model was induced by papain as described previously [17]. Briefly, a solution of 4% (w/v) papain solution in saline was sterilized and then injected into the right knee of rats on days 1, 4, and 7 of the experimental period. As a normal control, the same volume (20 *μ*L) of sterile saline was injected into the right knee of rats in the control group on the same days. Seven days after the last injection of papain, rats were injected intra-articularly with 0.01, 0.025 or 0.05% APS-3c (20 *μ*L) every 5 days for a period of 5 weeks as described previously [17]. Animals in the APS-3c group were injected with 20 *μ*L of 0.05% APS-3c. The normal control group and the OA model control group were injected with 20 *μ*L sterile saline. Animals were sacrificed at the end of the experiment and cartilage samples were removed from the tibial plateau in the right knee. Samples were fixed in 4% paraformaldehyde overnight, embedded in paraffin wax and then cut into 5 *μ*m sections perpendicular to the surface. Some sections were stained with haematoxylin-eosin (HE) and safranin O using standard protocols. The degree of OA was independently evaluated by two blinded observers according to the Mankin scoring system [25]. In Mankin scoring system, the structure of the cartilage, cell appearance, staining of the cartilage matrix by Safranin-O, tidemark, and pannus formation are evaluated. Each sample takes points in five different categories separately. By this system, total score changes between 0 and 14. Low scores show slight changes in articular cartilage where as high ones indicate severe osteoarthritis [25]. 

### 2.3. *In Vitro* Experiments

#### 2.3.1. Chondrocytes Culture and Treatment

Male Wistar rats (8 weeks old, Animal Center of Wuhan University) were housed under controlled temperature and lighting conditions with food and water. Articular cartilage isolated from femoral head cap pieces was aseptically dissected, and chondrocytes were obtained after digestion of cartilage fragments in 0.25% trypsin (w/v) for 30 min followed by about 6-7 h digestion in 0.2% collagenase II (w/v) in Dulbecco's modified Eagle's medium (DMEM) without serum. Six rat femoral heads would be needed to yield 10^6^ chondrocytes for the *in vitro* experiment. Chondrocytes were cultured at a density of 1 × 10^5^ cells/mL in DMEM with 10% fetal bovine serum. Experiments were performed with first-passage cultures. Chondrocytes were divided into five groups: (i) normal control group, chondrocytes without any treatments; (ii) OA model control group, chondrocytes treated with 20 ng/mL IL-1*β* (PeproTech, USA); (iii) APS-3c treatment groups, OA model chondrocytes treated with 20 ng/mL IL-1*β* and 2, 10, or 50 *μ*g/mL APS-3c, respectively. Chondrocytes were serum starved and pretreated with 2, 10, or 50 *μ*g/mL APS-3c alone for 4 h before being cotreated with different concentrations of APS-3c mentioned above and 20 ng/mL IL-1*β* for 24 h. In order to investigate whether different concentrations of APS-3c themselves presented cytotoxicity on chondrocytes (detection of cell viability), normal chondrocytes were only treated with 2, 10, or 50 *μ*g/mL APS-3c, respectively, for 24 h, and then followed by assay of cell viability. These experiments were performed in triplicate and the results are provided as mean values from three independent experiments.

#### 2.3.2. Assay of Cell Viability

After chondrocytes had been cultured in 96-well plate (1 × 10^4^ cells/well) for 24 h, the medium replaced with DMEM containing 5 mg/mL 3-(4, 5-dimethyl-2-thiazolyl)-2,5-diphenyl-2 h-tetrazolium bromide (MTT) and incubated for 4 h at 37°C. Formazan products were dissolved in 100 *μ*L dimethylsulphoxide (DMSO) and absorbance was measured at 570 nm using a microplate reader (Shimadzu, Kyoto, Japan).

#### 2.3.3. Detection of Apoptosis

Chondrocytes were resuspended in 200 *μ*L HEPES buffer, stained with 5 *μ*L Annexin-V-FITC and 10 *μ*L PI for 15 min at room temperature in the dark. After incubation, 200 *μ*L HEPES buffer was added, the cells were measured on EPICS ALTRAII flow cytometry (Beckman, USA) and analyzed with Multi-cycle software (Phoenix Flow Systems, USA). Results were expressed as percentage (PI negative and Annexin-V positive) apoptotic cells. All experiments were performed in triplicate.

#### 2.3.4. Assay of Glycosaminoglycan (GAG) Contents in Cell Supernatants

The dimethylmethylene blue (DMMB) spectrophotometric assay was used to determine GAG contents in cell supernatants [26]. Cell supernatants were digested in 0.5 mg/mL papain for 2 hours at 65°C. Following digestion, they were centrifuged at 1,500 g for 8 min. Then, DMMB solution (Sigma, St. Louis, MO, USA) was added to cell supernatants. The absorbance was measured at 595 nm using a UV-1601 spectrophotometer (Shimadzu, Kyoto, Japan). Chondroitin-6-sulfate from shark cartilage (Sigma, St. Louis, MO, USA) was used to construct the standard curve, and the GAG content was calculated. DNA content was measured using a Hoechst dye and used for normalization [27]. We found that APS-3c did not interact with DMMB and interfere with the GAG quantitation by setting the APS-3c control group in the DMMB spectrophotometric assay.

#### 2.3.5. Chondrocytes Proteoglycan Synthesis

The incorporation of ^35^S-sulfate into PG by articular chondrocytes was assessed as described by van der Kraan et al. [28]. Chondrocytes were labeled with 10 *μ*Ci/mL ^35^S-sulfate (Amersham, Les Ulis, France) for the last 6 h of the experimental period. After incubation, the media and cell extracts were incubated overnight at room temperature in 0.5% cetylpyridinium chloride. GAG was dissolved in Soluene-350 (Packard, Rungis, France), and the radioactivity of  ^35^S-sulfate incorporated was measured by liquid scintillation counting (Packard, Rungis, France). For each experiment, the amount of DNA was measured in sister flasks. Results were calculated as the mean ± SEM disintegrations per minute/10 *μ*g DNA in 6 similarly treated wells. 

#### 2.3.6. DNA Microarray Experiments

Total RNA was extracted from chondrocytes using TRIzol (Invitrogen, Carlsbad, CA, USA) following the manufacturer's instruction. The concentration and purity of RNA were measured using the NanoDrop method (3300 NanoDrop Analyzer, Thermo Scientific, USA). Agilent Whole Rat Genome 4 × 44 K Microarray (Agilent Technologies, Palo Alto, CA, USA) was manufactured by Agilent. Updated content provided full coverage of rat genes and transcripts. Sample labeling and array hybridization were performed according to the Agilent One-Color Microarray-Based Gene Expression Analysis protocol. 

#### 2.3.7. Reverse Transcription Polymerase Chain Reaction (RT-PCR)

Equal amounts (1 *μ*g) of total RNA were reverse transcribed into cDNA using a first-strand cDNA synthesis kit (TransGen Biotech Co., Ltd.). The RNA was assayed for GAPDH, aggrecan, collagen type II a 1 (Col2a1), O-xylosyltransferase I (XylT-I), *β*1, 4-galactosyltransferase 7 (GalT-I), *β*1,3-galactosyltransferase 6 (GalT-II), *β*1,3-glucuronosyltransferase I (GlcAT-I), N-acetylgalactosaminyltransferase 1 (CSGalNAc-T1), aggrecanase-1, aggrecanase-2, and MMP-3 messenger RNA (mRNA) using one-step RT-PCR reactions in a 2720 Thermal Cycler (Applied Biosystems, USA). Primers were designed according to previously described by Arner et al. and Huang et al. [7, 8]. Promega Pfu DNA Polymerase (Promega, CA, USA) was used for PCR. The annealing temperature and cycle number for each set of primers for the genes used in this experiment are exhibited in Table 1. The PCR was initiated with a denaturation at 95°C for 5 min, followed by the optimal number of cycles of 95°C for 5 s, annealing for 20 s and an extension stage at 72°C for 20 s. Lastly, the cDNA went through an extension stage at 72°C for 5 min, and the products were held at 4°C until gel electrophoresis of the products. Each PCR sample was separated on a 2% agarose gel and electrophoresed for 40 min. Each gel was photographed and analyzed using G: BOX Syngene. For each gene of interest, the amplicon density levels were divided by the corresponding value for the gene GAPDH from the same sample. All samples from a single experiment were run on the same plate, generally using four replicate determinations per sample. Each gel was analyzed 3 times with the Syngene Systems.

#### 2.3.8. Western Blotting Analysis

After treatment, cells were washed with ice-cold PBS and lysed in the lysis buffer (150 mM NaCl, 50 mM Tris HCl, 1 mM EDTA, 0.5% NP-40, and PH 7.5). Cell lysate was centrifuged, and the supernatant protein was quantified using the BCA protein assay kit (Bio-Rad, CA, USA). Equal amounts of protein (40 ug) were mixed with 2 × Laemmli sample buffer (Sigma-Aldrich, MO, USA) and separated on SDS-PAGE (5 and 12% gels) under reducing conditions and transferred to polyvinylidene difluoride membranes. Membranes were blocked and probed with anti-rat XylT-I (1 : 200), GlcAT-I (1 : 200), and *β*-actin (1 : 2000) primary antibodies (Santa Cruz, CA, USA) at 4°C overnight, then incubated with horseradish peroxidase-conjugated secondary antibodies (1 : 2000), and followed with visualization by the Chemiluminescence Luminol Reagent (Santa Cruz, CA, USA) with signals captured on film.

### 2.4. Statistical Analysis

Data were expressed as mean ± SEM. Statistical analysis of quantified data was analyzed by analysis of variance (ANOVA). The results were considered of statistical significance when the *P* values were <0.05.

## 3. Results

### 3.1. Protective Effects of APS-3c on Rat OA Cartilage *In Vivo*

**Figure 1:**
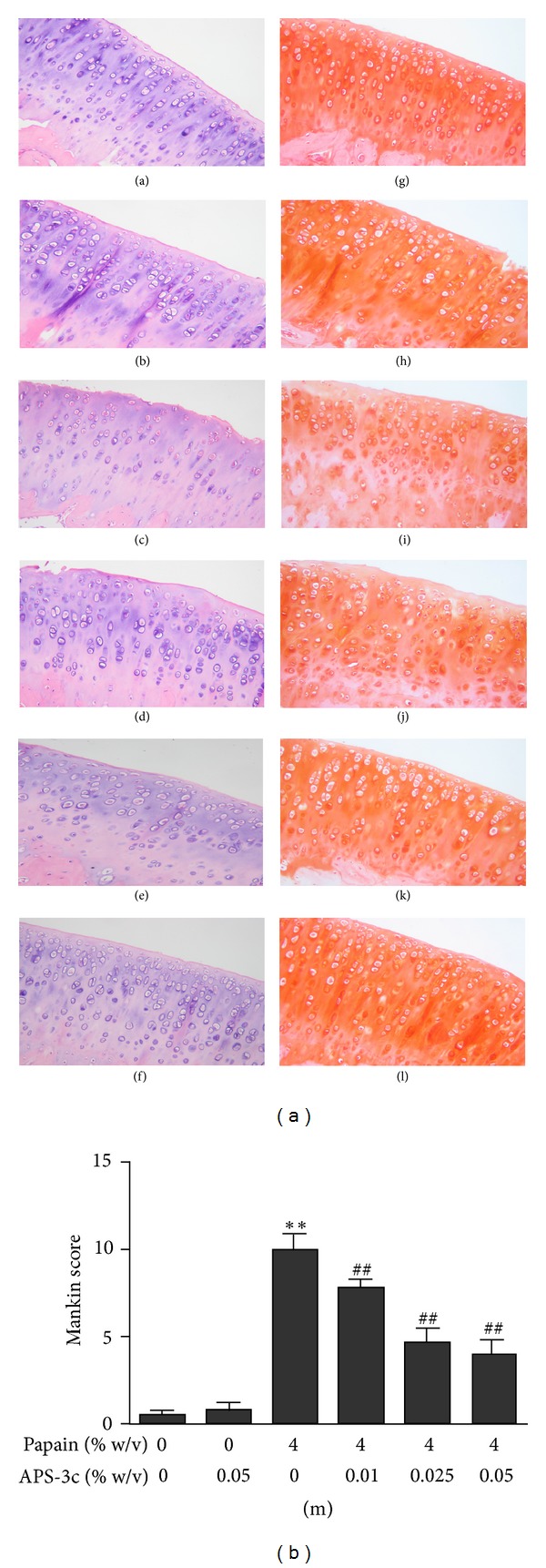
Effects of *Angelica sinensis* polysaccharides (APS-3c) on histological changes in cartilage as assessed by haematoxylin-eosin staining ((a), (b), (c), (d), (e) and (f), original magnification ×200) and Safranin O staining ((g), (h), (i), (j), (k) and (l), original magnification ×200) on rat osteoarthritis (OA) induced by papain. ((a) and (g)) Normal control group; ((b) and (h)) APS-3c control group: rats treated with 0.05% APS-3c; ((c) and (i)) OA model control group: papain-induced OA rats treated with sterile saline; ((d) and (j)) OA rats treated with 0.01% APS-3c; ((e) and (k)) OA rats treated with 0.025% APS-3c; ((f) and (l)) OA rats treated with 0.05% APS-3c every 5 days for 5 weeks. (m) Mankin score. Values represent Mean ± SEM of eight different simples. **P < 0.01 *versus* normal control group, ^##^ *P* < 0.01 *versus* OA model control group.

Cartilage in normal control rats had a normal chondrocytes structure, an intact surface, a tidemark, and typical staining of the matrix with Safranin O (Figures 1(a) and 1(g)). The APS-3c control group exhibited similar results (Figures 1(b) and 1(h)). Cartilage from the OA model control group showed marked morphological changes, including irregular surface, fibrillation, roughing, derangement of the cells in tangential zone, reduction in staining and multilayered tidemark zone (Figures 1(c) and 1(i)). In view of the Safranin O staining apparent in Figure 1(i), one had the impression that spontaneous repair is occurring. This may be due to the young age of the rats. The OA groups treated with 0.01, 0.025, and 0.05% APS-3c showed an intact surface and ordered layers (Figures 1(d), 1(e), and 1(f)). Matrix staining was better in OA rats treated with 0.01, 0.025 and 0.05% APS-3c than the OA model control group (Figures 1(j), 1(k), 1(l)). Treatment with APS-3c significantly decreased the total Mankin score for OA cartilage in a concentration-dependent manner (Model control group: 9.8 ± 0.97, APS-3c treatment group: 7.6 ± 0.53, 4.5 ± 0.85, 3.9 ± 0.79, all *P* < 0.01; *n* = 8), but had no significant effect on normal cartilage (APS-3c control group: 0.7 ± 0.46, normal control group: 0.4 ± 0.26, *P* > 0.05; *n* = 8) (Figure 1(m)). It suggested that APS-3c promoted the restoration of cartilage matrix and had a protective effect on cartilage degradation in rat OA model.

### 3.2. APS-3c Enhanced IL-1*β*-Stimulated Chondrocytes Proteoglycan Synthesis and Had No Influence on the Viability of Chondrocytes and the Expression of Type II Collagen *In Vitro*

**Figure 2:**
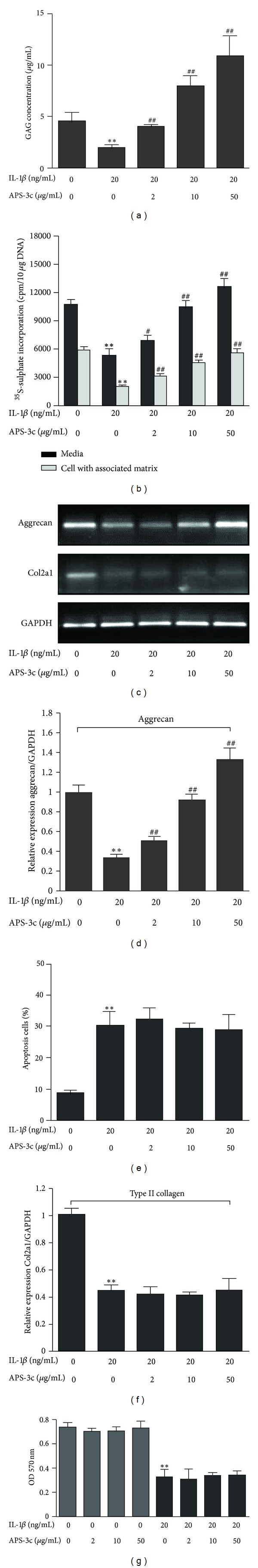
Effect of *Angelica sinensis* polysaccharides (APS-3c) on proteoglycan synthesis, cell proliferation, and cell apoptosis of rat interleukin-1-beta- (IL-1*β*-) stimulated chondrocytes. (a) Effect of APS-3c on glycosaminoglycan (GAG) contents in cell supernatants of rat IL-1*β*-stimulated chondrocytes. (b) Effect of APS-3c on proteoglycan synthesis of rat IL-1*β*-stimulated chondrocytes. Chondrocytes were incubated for the final 6 h with 10 *μ*Ci/mL ^35^S-sulfate in DMEM supplemented in the presence or absence of stimulants. After incubation, the media and cell extracts were incubated overnight at room temperature in 0.5% cetylpyridinium chloride. GAG was dissolved in Soluene-350 and the radioactivity of  ^35^S-sulfate incorporated was measured by liquid scintillation counting. For each experiment, the amount of DNA was measured in sister flasks. Results were calculated as the mean ± SEM cell or medium disintegrations per minute/10 *μ*g DNA in 6 similarly treated wells. (c) RT-PCR of aggrecan, Col2a1, and GAPDH (from top to bottom) mRNA in response to various concentrations of APS-3c. ((d) and (f)) The resulting data were expressed and illustrated as a ratio of the normal control. Integrated density values for polymerase products were normalized to the values for GAPDH. (e) Effect of APS-3c on apoptosis rate of rat IL-1*β*-stimulated chondrocytes. Chondrocytes were incubated with APS-3c alone for 4 h and then cotreated with IL-1*β* and APS-3c for 24 h. After being stained with annexin-V-fluoresceine isothiocyanate (FITC) and propidium iodide (PI), chondrocytes were measured on flow cytometry and analyzed with Multi-cycle software. (g) Effect of APS-3c on proliferation of rat chondrocytes in the presence or absence of IL-1*β*. Values represent Mean ± SEM of six different simples. **P < 0.01 *versus* normal control. ^#^ *P* < 0.05, ^##^ *P* < 0.01 *versus* IL-1*β*-stimulated control.

 GAG contents in cell supernatants were first detected by the DMMB spectrophotometric assay. The levels of GAG were markedly lower in the IL-1*β*-stimulated control (1.93 ± 0.29 *μ*g/mL) compared with the normal control (4.55 ± 0.87 *μ*g/mL). This suggested that IL-1*β* significantly decreased the GAG contents in cell supernatants. However, the levels of GAG were higher in the 2 *μ*g/mL APS-3c group (3.98 ± 0.16 *μ*g/mL), 10 *μ*g/mL APS-3c group (7.98 ± 0.97 *μ*g/mL), and 50 *μ*g/mL APS-3c group (10.89 ± 1.91 *μ*g/mL) than that in the IL-1*β*-stimulated control (all *P* < 0.01; *n* = 6). APS-3c increased the levels of GAG inhibited by IL-1*β* in cell supernatants (Figure 2(a)).

To determine whether the increase of GAG contents mediated by APS-3c was due to increased synthesis of PG or not, PG synthesis was examined by ^35^S-sulfate radiolabeling. IL-1*β* significantly decreased the amount of newly synthesized PG in chondrocytes with associated matrix and in the media (both *P* < 0.01; *n* = 6). However, APS-3c concentration dependently enhanced the amount of newly synthesized PG in chondrocytes with associated matrix and in the media at 2~50 *μ*g/mL (all *P* < 0.01 or 0.05; *n* = 6). It suggested that the effect of APS-3c on IL-1*β*-stimulated chondrocytes was mainly reflected in PG synthesis (Figure 2(b)).

We also performed RT-PCR analysis to detect the gene expression of aggrecan and Col2a1. Chondrocytes stimulated with IL-1*β* (20 ng/mL) showed a decrease in the mRNA expression of aggrecan and Col2a1 (both *P* < 0.01; *n* = 4). Treatment with APS-3c improved the gene expression of aggrecan in a concentration-dependent manner compared with IL-1*β*-stimulated control (all *P* < 0.01; *n* = 4). However, treatment with 2, 10 and 50 *μ*g/mL concentrations of APS-3c did not show any significant change on Col2a1 mRNA expression in rat chondrocytes stimulated with IL-1*β* (Figures 2(c), 2(d), and 2(f)).

Monolayer-cultured chondrocytes viability in the APS-3c group was no significantly lower than that in the normal control at 2, 10, and 50 *μ*g/mL concentrations. It showed that different concentrations of APS-3c from 2~50 *μ*g/mL have no cytotoxicity on normal chondrocytes. IL-1*β* significantly inhibited chondrocytes proliferation in the IL-1*β*-stimulated control compared with the normal control (*P* < 0.01; *n* = 4). However, 2, 10, and 50 *μ*g/mL APS-3c had no effect on chondrocytes proliferation in the presence or absence of 20 ng/mL IL-1*β* (Figure 2(g)).

Chondrocytes apoptosis was identified by FITC-Annexin V/PI double-labeled assay. IL-1*β* significantly increased the percentage of apoptotic chondrocytes in the IL-1*β*-stimulated control (30.2 ± 4.1%) compared with the normal control (8.3 ± 1.2%). However, 2, 10, and 50 *μ*g/mL APS-3c also had no influence on chondrocytes apoptosis compared with the IL-1*β*-stimulated control (Figure 2(e)).

### 3.3. Gene Expression Profile by Microarray Analysis

The results mentioned above showed that APS-3c improved PGs synthesis inhibited by IL-1*β*. To elucidate the possible molecular mechanism, we scanned the global expression of rat chondrocytes with Agilent Rat Whole Genome 4 × 44 K microarray. A total of 8 samples (2 normal controls, 3 IL-1*β*-stimulated controls, and 3 APS-3c 50 *μ*g/mL treatment groups) were analyzed as described in detail in Section 2. Setting a filter to a fold change (FC) equal to ±2, we found that 264 (1%) and 242 (1%) genes were significantly up- or downregulated in 50 *μ*g/mL APS-3c treatment group *versus *IL-1*β*-stimulated control group, respectively. 

We mainly focused on specific genes involved in PGs metabolism.  Table 2 showed the 26 genes about PGs metabolism in chondrocytes. Among these genes, we found that the gene expression of aggrecan, XylT-1, GalT-I, GlcAT-I, and chondroitin sulfate synthase 1 (Chsy1) was upregulated at least 2-fold and the gene expression of GalT-II, Csgalnact1, Csgalnact2, and biglycan (Bgn) were upregulated at least-1.5 fold in 50 *μ*g/mL APS-3c treatment group *versus* IL-1*β*-stimulated control group. However, the gene expression of matrix-degrading enzymes, including aggrecanase-1, aggrecanase-1, MMP-2, MMP-3, MMP-9, and MMP-13, did not appear to be significantly changed. The results suggested that the protective effects of APS-3c on IL-1*β*-stimulated chondrocytes may be mediated by these genes involved in PGs synthesis.

### 3.4. RT-PCR Analysis

**Figure 3:**
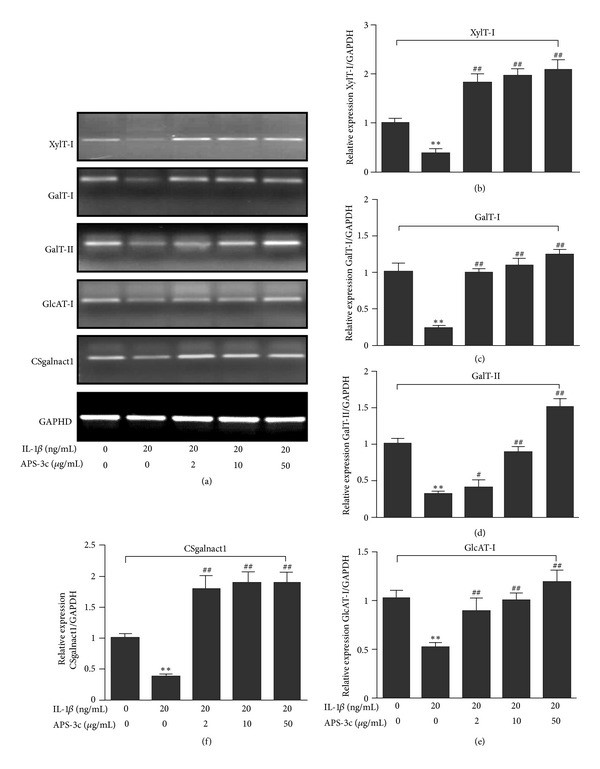
Effect of *Angelica sinensis* polysaccharides (APS-3c) on the expression of chondrocytes glycosyltransferases genes involved in glycosaminoglycan synthesis of rat interleukin-1-beta- (IL-1*β*-) stimulated chondrocytes. (a) RT-PCR of O-xylosyltransferase I (XylT-I), *β*1,4-galactosyltransferase 7 (GalT-I), *β*1,3-galactosyltransferase 6 (GalT-II), *β*1,3-glucuronosyltransferase I (GlcAT-I), N-acetylgalactosaminyltransferase 1 (Csgalnact-1), and GAPDH (from top to bottom) mRNA in response to various concentrations of APS-3c. Integrated density values for polymerase products were normalized to the values for GAPDH. ((b), (c), (d), (e), and (f)). The resulting data were expressed and illustrated as a ratio of the normal control. Values represent Mean ± SEM of four different simples. **P < 0.01 *versus* normal control. ^#^ *P* < 0.05, ^##^ *P* < 0.01 *versus* IL-1*β*-stimulated control.

In order to confirm the results of Microarray and the regulatory role of APS-3c on PGs synthesis progress, we performed RT-PCR analysis to detect the expression of specific genes involved in PGs metabolism. We determined the expression levels of five GTs genes (XylT-I, GalT-I, GalT-II, GlcAT-I, and CSgalnact-1) involved in GAG synthesis with RT-PCR analysis. As shown in Figure 3, treatment with IL-1*β* caused significant decrease in XylT-I, GalT-I, GalT-II, GlcAT-I, and CSgalnact-1 mRNA expressions, whereas coincubation with 2, 10, and 50 *μ*g/mL concentrations of APS-3c appeared to approve these genes expression in a concentration-dependent manner compared with IL-1*β*-stimulated control (all *P* < 0.01; *n* = 4). The results corroborated the analysis of DNA Microarray, in which APS-3c mainly upregulated the gene expression of GTs involved in GAG synthesis. Combined with the result of the aggrecan gene expression, we speculate that APS-3c can improve PGs synthesis on rat IL-1*β*-stimulated chondrocytes, which is due to the promotion of these genes involved in PGs synthesis progress to promote cartilage repair (Figure 3). 

**Figure 4:**
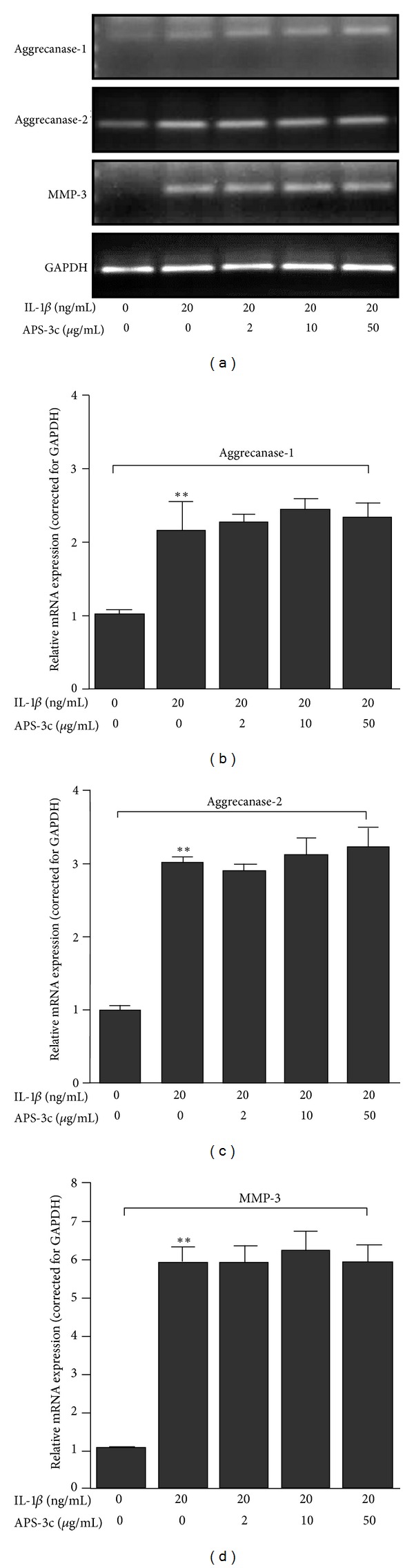
Effect of *Angelica sinensis* polysaccharides (APS-3c) on the expression of chondrocytes specific genes involved in proteoglycan-degrading enzymes of rat interleukin-1-beta- (IL-1*β*-) stimulated chondrocytes. (a) RT-PCR of aggrecanase-1, aggrecanase-2, MMP-3 and GAPDH (from top to bottom) mRNA in response to various concentrations of APS-3c. Integrated density values for polymerase products were normalized to the values for GAPDH. ((b), (c), and (d)). The resulting data were expressed and illustrated as a ratio of the normal control. Values represent Mean ± SEM of four different simples. **P < 0.01 *versus* normal control. ^##^ *P* < 0.01 *versus* IL-1*β*-stimulated control.

Then we determined the gene expression levels of PGs-degrading enzymes with RT-PCR analysis. As shown in Figure 4, chondrocytes stimulated with IL-1*β* (20 ng/mL) showed a several-fold increase in the expression of aggrecanase-1, aggrecanase-2, and MMP-3 (all *P* < 0.01; *n* = 4). However, treatment with 2, 10, and 50 *μ*g/mL concentrations of APS-3c did not show any significant change on aggrecanase-1, aggrecanase-2, and MMP-3 mRNA expressions in rat chondrocytes stimulated with IL-1*β* (Figure 4).

### 3.5. Western Blotting Analysis

**Figure 5:**
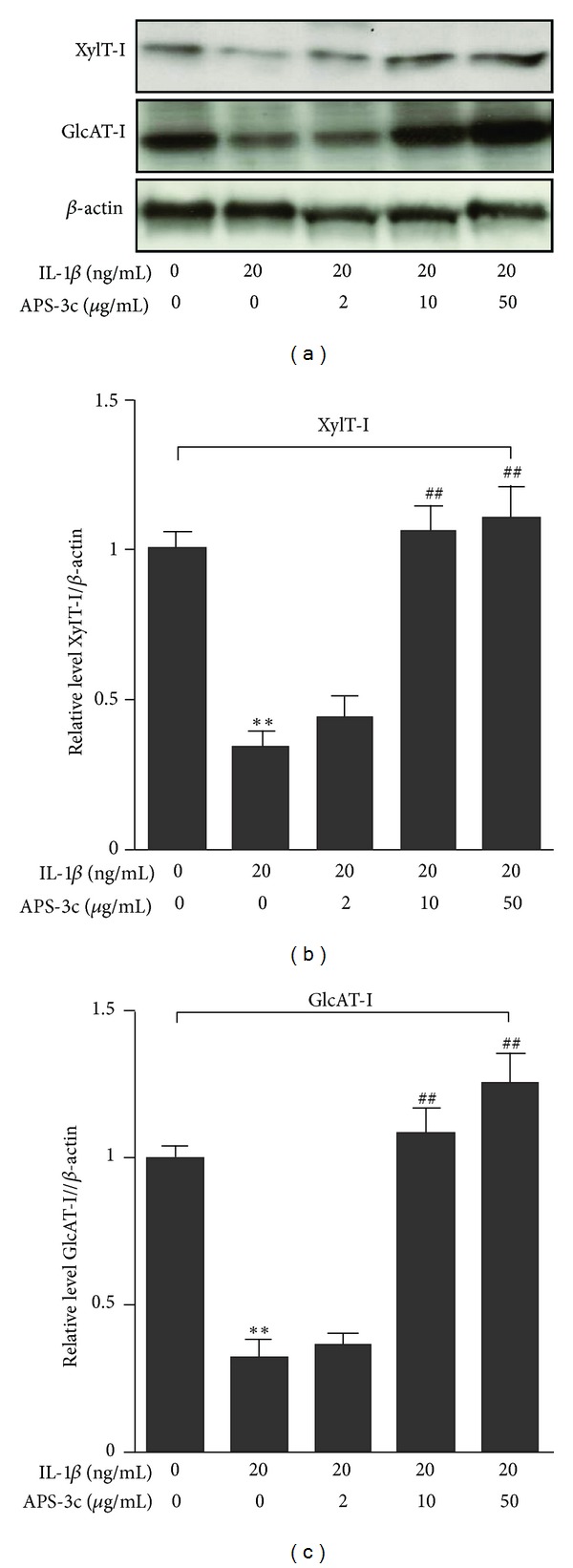
Effect of *Angelica sinensis* polysaccharides (APS-3c) on the protein expression of O-xylosyltransferase I (XylT-I), *β*1,3-glucuronosyltransferase I (GlcAT-I) and *β*-actin in rat interleukin-1-beta-(IL-1*β*-) stimulated chondrocytes. Expressions of proteins determined by western blotting. (a) Western blotting of O-xylosyltransferase I (XylT-I), *β*1,3-glucuronosyltransferase I (GlcAT-I) and *β*-actin (from top to bottom) protein in response to various concentrations of APS-3c. ((b) and (c)) Relative level of XylT-I and GlcAT-I normalized to *β*-actin and compared with normal control, quantitatively analyzed by Kodak Digital Science 1D software (Eastman Kodak, Rochester, NY, USA) and expressed as mean optical density. Values represent mean ± standard error of the mean of three different simples. **P < 0.01 versus normal control. ^##^ *P* < 0.01 versus IL-1*β*-stimulated control.

In order to further confirm the expression of related protein, we detected XylT-I and GlcAT-I level by western blotting. The result also suggested that IL-1*β* could repress the XylT-I and GlcAT-I protein expression (*P* < 0.01; *n* = 4). However, 10 and 50 *μ*g/mL concentrations of APS-3c could promote the XylT-I and GlcAT-I protein expression in rat IL-1*β*-stimulated chondrocytes (both *P* < 0.01; *n* = 4) (Figure 5).

## 4. Discussion 

Our results showed that APS-3c had a protective effect on both articular cartilage in rat OA model and cultured rat chondrocytes induced by IL-1*β*. Histological analysis showed that APS-3c lowered the Mankin score of OA cartilage and promoted the restoration of cartilage matrix. The *in vitro* results revealed that APS-3c enhanced GAG biosynthesis and upregulated the expression of aggrecan gene. This is the first study on the effects of APS-3c on OA. 

With increasing age, the prevalence of OA increases and the efficacy of articular cartilage repair decreases. The articular cartilage ECM is composed of collagenous proteins, mainly type II, glycoprotein such as link protein, and proteoglycans such as aggrecan. As chondrocytes age, they synthesize smaller, less uniform aggrecan molecules and less functional link proteins, their mitotic and synthetic activity decline, and their responsiveness to anabolic mechanical stimuli and growth factors decreases. These alterations in material properties are associated with occurrence and progression of the disease [29]. PG comprises GAG chains covalently linked to a core protein [6]. The biosynthesis of GAG chains is initiated by the formation of a tetrasaccharide, GlcA*β*1, 3Gal*β*1, 3Gal*β*1, and 4Xyl*β*-O attached to the hydroxyl group of specific serine residues of different core proteins. This linkage tetrasaccharide is formed by a series of monosaccharides derived from UDP sugars catalyzed by XylT-I, GalT-I, GalT-II, and GlcAT-I and serves as a primer for CS chain elongation [11]. CS consists of repeating disaccharide units of N-acetylgalactosamine (GalNAc) and glucuronic acid (GlcA) residues catalyzed by CSGalNAc-T1 and chondroitin sulfate glucuronyltransferase (CSGlcA-T) [30]. In cartilage, a large chondroitin sulfate proteoglycan (CSPG) aggrecan, which is thought to contain approximately one hundred CS chains, forms PG aggregates together with hyaluronan and link protein-1. The aggregates contribute to water retention and resistance to compression [31]. 

Recent research showed that the expression of XylT-I, which initiates PG-GAG chain synthesis by transferring the first sugar residue (xylose) to specific serine residues of PG core proteins, was downregulated in cartilage isolated from an animal model of arthritis and was associated with reduced PG synthesis. GalT-I catalyzes the transfer of a Gal residue provided by UDP-Gal onto Xyl, a key step in the synthesis of the linkage region of GAG chains [32]. Meanwhile, some studies indicate that GlcAT-I, a rate-limiting enzyme in GAG biosynthesis, completes the synthesis of the common linker region of GAG by transferring glucuronic acid to the core protein Gal-Gal-Xyl-O-Ser trisaccharide. It was reported that IL-1*β* could repress GlcAT-I and XylT-I gene expression and activity and reduce GAG biosynthesis observed in rat chondrocytes [12, 33]. CSGalNAcT1 has one glycosyltransferase domain, exhibits GalNAc transfer activity in both the initiation and elongation processes, and is thought to be responsible for the addition of the first GalNAc residue to the tetrasaccharide chain [34]. In this research, the genome Microarray analysis showed that IL-1*β* downregulated the expression of the specific genes which were involved in PG synthesis, whereas treatment with APS-3c could improve the inhibition action of IL-1*β*. The RT-PCR analysis further confirmed that IL-1*β* repressed the mRNA expressions of aggrecan, XylT-I and GlcAT-I as previous described [12, 33], as well as three other GTs, including GalT-I, GalT-II, and CSGalNAc-T1, which were first observed. However, we showed that APS-3c up-regulated the mRNA expression of these genes which were involved in PGs synthesis. The results of western blotting also confirmed that IL-1*β* repressed the protein expressions of XylT-I and GlcAT-I. However, we found that APS-3c promoted the protein expression of XylT-I and GlcAT-I in rat IL-1*β*-stimulated chondrocytes. Meanwhile, we also found that APS-3c promoted PGs synthesis inhibited by IL-1*β* in rat chondrocytes, which was confirmed by both the GAG contents and the ^35^S-sulfate incorporation assay.

The imbalance between biosynthesis and degradation of PGs is the salient characteristic of OA [35]. Many researchers studying alterations in cartilage PGs metabolism in OA have also focused on the mechanism of PGs degradation. Accordingly, the enzymes that degrade PGs have been studied as potential targets for OA therapy [36, 37]. Two cartilage aggrecanases, aggrecanase-1 and -2, have been found to be responsible for aggrecan cleavage during cartilage degradation and cleave at the Glu373-Ala374 bond in the interglobular domain (IGD) [7, 8]. MMP-3 not only cleaves aggrecan at the Asn341-Phe342 site within the IGD, but also activates the proforms of several MMPs and contributes to the activation of aggrecanase-2 [9, 10]. IL-1*β* can promote PGs breakdown by activating aggrecanase-1, aggrecanase-2, and MMP-3 [38]. Our findings corroborated the abovementioned effects of IL-1*β* in cultured rat IL-1*β*-stimulated chondrocytes. The genome Microarray analysis suggested that IL-1*β* up-regulated the expression of the genes which were involved in PGs degradation, whereas treatment with APS-3c did not show any significant changes on the expression of these genes in rat chondrocytes stimulated with IL-1*β*. The RT-PCR analysis further confirmed that APS-3c had no effect on the mRNA expression of aggrecanase-1, aggrecanase-2, and MMP-3 which were involved in PGs degradation. 

The loss of PGs also arises from an altered anabolic response of the chondrocyte, which is not only critical for normal cartilage homeostasis but also of potential significance during metabolic changes in OA, especially when chondrocytes attempt to repair the degraded ECM in the early stages of OA [12]. Young rats possessed a strong ability to metabolize PGs in the articular cartilage. In addition, in order to exclude the impact of gender, we chose the 8-week-old male Wistar rat to establish the animal model of OA and for chondrocytes culture. Papain that is one of the proteolytic enzyme is often used for induction of experimental OA [39–41]. Findings observed after papain injection seem to be consistent with early osteoarthritic changes [39]. In view of the Safranin O staining apparent in Figure 1(i), one had the impression that spontaneous repair was occurring. This may be due to the young age of the rats. However, we found that APS-3c promoted the restoration of cartilage matrix and had a protective effect on cartilage degradation in rat OA model. Meanwhile, there is no guarantee that results observed with young cartilage will be totally repeated in the adult.

The chondrocyte is the only resident cell found in cartilage and is responsible for maintaining matrix integrity, pathological cascade process, and tissue homeostasis. The tissue damage stimulates a chondrocytic synthetic and proliferative response that may maintain or even restore the articular cartilage [42]. The apoptosis-positive cells might be due to a protection mechanism after sublethal injury, in particular, represented by an increased survival of chondrocytes that are able to participate in the repair process [43, 44]. Beyond the compensation capability of chondrocytes on OA process, apoptosis cells become the main source of various catabolic factors, such as proteases, proinflammatory mediators, nitric oxide (NO), and oxygen radicals [45]. Therefore, chondrocytes survival or apoptosis is important in the pathogenesis of OA. IL-1*β* can inhibit chondrocytes proliferation and promote chondrocytes apoptosis by activating nuclear factor-*κ*B (NF-*κ*B) signal pathway [14]. In the present study, we found that APS-3c had no influence on chondrocytes apoptosis and proliferation treatment with IL-1*β* *in vitro.* Although IL-1*β* plays a central role in the pathogenesis of OA, IL-1*β*-stimulated normal chondrocyte is different from OA chondrocyte. While IL-1*β* exposure occurs in OA, the chondrocytes are also usually considered to be phenotypically distinct from normal chondrocytes. Further studies are needed to elucidate the precise mechanism of APS-3c on PGs synthesis in OA chondrocytes.

These results suggested that the protective effect of APS-3c on rat IL-1*β*-stimulated chondrocytes was not promotion of proliferation, attenuation of apoptosis, or inhibition of the expression of PGs-degrading enzymes, but promotion of PGs synthesis, which was due to the promotion of the expression of aggrecan gene and GTs gene involved in PGs synthesis. The component sugars of APS-3c were Glu, Gal, Ara, Rha, Man, and Xyl. In previous research, we also observed that APS had a repeating unit consisting of (1→4)-*α*-D-glucopyranosyl residue and (1→6)-*α*-D-glucopyranosyl residue [46]. Moreover, Yamada et al. [47] reported that APS was a (1→4)-*α*-D-glucan having side chains at O-6 of the glucosyl residues of the main chain. So, we speculated that the protective effect of APS-3c on rat IL-1*β*-stimulated chondrocytes might be attributed to its consistent of six monosaccharides and the similar primary structure as GAG. The works to further verify the relationship between APS-3c and PGs synthesis by using transfection or siRNA are needed to pursue in future. In addition, our previous study showed that ferulic acid, the other natural component of *Angelica sinensis*, can repress the expression of MMPs to prevent ECM degradation processes, promote chondrocytes proliferation, and reduce chondrocytes apoptosis in *in vivo* and *in vitro* OA model [18]. The combination of these effects of two components could be better to elucidate the pharmacological mechanism of *Angelica sinensis* on OA. It also provided a possible scientific basis for the multitarget treatment of traditional Chinese herbs.

## 5. Conclusions

In conclusion, we observed the protective effects of APS-3c on OA animal model *in vivo* and rat IL-1*β*-stimulated chondrocytes *in vitro*. The findings indicate that APS-3c can improve PGs synthesis of chondrocytes, which is due to the promotion of the expression of aggrecan gene, and GTs gene involved in PGs synthesis. Our findings provide a novel understanding of the pharmacological effects of APS-3c and suggest the clinical relevance of APS-3c in the prospective of future alternative medical treatment for OA.

## Figures and Tables

**Table 1:** Primer sequence and the optimal PCR conditions.

Gene	Accession number	Sequence	Tm (°C)	Cycle number	Product size (bp)
Aggrecan	NM_022190	F: 5′-CTAGCTGCTTAGCAGGGATAACG-3′	58	20	108
		R: 5′-TGACCCGCAGAGTCACAAAG-3′			
Col2a1	NM_012929	F: 5′-GAGTGGAAGAGCGGAGACTACTG-3′	57	15	81
		R: 5′-CTCCATGTTGCAGAAGACTTTCA-3′			
XylT-I	XM_341912	F: 5′-TTCACTGGGCGAGAGTGCAAGG-3′	57	25	346
		R: 5′-TGCCTGGGGCTTTTTCCCGA-3′			
GalT-I	NM_001031661	F: 5′-CATGCACGATGTGGACCTAC-3′	59	25	147
		R: 5′-TGTTTGGACAGCAGCAGAAT-3′			
GalT-II	NM_001106699	F: 5′-GCTTGGCAACTCTGCGACTA-3′	57	25	135
		R: 5′-GCCCAGCGATACATCTTCAC-3′			
GlcAT-1	NM_001128184	F: 5′-CACACACCTGGCTGTCCTTA-3′	59	25	147
		R: 5′-GAGTCCTTCTCTCCCCCTACA-3′			
CSgalnact-1	NM_001107309	F: 5′-TGGAAGGGAAGTAATGTCGT-3′	51	25	159
		R: 5′-ATGATGGCCGTAGATTATGC-3′			
Aggrecanase-1	NM_023959	F: 5′-TCGCTTCGCTGAGTAGATTCGT-3′	55	25	141
		R: 5′-TTCGGATGCTTGGATGCTTAA-3′			
Aggrecanase-2	NM_198761	F: 5′-CTGCGCTGTGATTGAAGATGAT-3′	59	20	158
		R: 5′-TGCTGGTAAGGATTGAAGACATT-3′			
MMP-3	NM_133523	F: 5′-TGGCAGTGAAGAAGATGCTG-3′	61	25	167
		R: 5′-GCTTCCCTGTCATCTTCAGC-3′			
GAPDH	NM_017008	F: 5′-GGCTCTCTGCTCCTCCCTGT-3′	59	20	123
		R: 5′-GTAACCAGGCGTCCGATACGGC-3′			

PCR: polymerase chain reaction; Col2a1: collagen type II a 1; XylT-I: xylosyltransferase I; GalT-I: *β*1,4-galactosyltransferase 7; GalT-II: *β*1,3-galactosyltransferase 6; GlcAT-1: *β*1,3-glucuronosyltransferase I; CSgalnact-1: N-acetylgalactosaminyltransferase 1; MMP-3: matrix metallopeptidase 3; GAPDH: glyceraldehyde-3-phosphate dehydrogenase.

**Table 2:** Concurrently expressed 26 genes involved in PGs metabolism of chondrocytes with Agilent Microarray Analysis.

Probe ID	Description	Genebank	Expression level			Ratio APS-3c treatment/ Model control
			Normal control	Model control	APS-3c treatment	
			IL-1*β*(−) APS-3c(−)	IL-1*β*(−) APS-3c(+)	IL-1*β*(+) APS-3c(+)	
A_43_P12154	Aggrecan (Acan)	NM_022190	463	62	382	6.16
A_43_P16196	Xylosyltransferase I (XylT-1)	XM_341912	177	75	194	2.59
A_43_P12206	xylosyltransferase II (Xylt2)	NM_022296	429	285	301	1.06
A_44_P363522	1,4-galactosyltransferase, polypeptide 7 (GalT-I)	NM_001031661	1256	648	1370	2.11
A_44_P384798	1,3-galactosyltransferase, polypeptide 6 (GalT-II)	NM_001106699	212	125	197	1.58
A_44_P532027	Beta-1,3-glucuronyltransferase 3 (GlcAT-I)	NM_001128184	4770	2031	5461	2.69
A_42_P573352	CS N-acetylgalactosaminyltransferase 1 (Csgalnact1)	NP_001100779	1274	694	1191	1.72
A_44_P102489	CS N-acetylgalactosaminyltransferase 2 (Csgalnact2)	NM_001106616	1929	1290	2016	1.56
A_44_P412933	Chondroitin polymerizing factor (Chpf)	NM_001005906	25074	23983	26080	1.09
A_44_P243367	Chondroitin sulfate synthase 1 (Chsy1)	NM_001106268	119	55	116	2.11
A_44_P414919	Chondroitin sulfate synthase 3 (Chsy3)	XM_225912	195	226	242	1.07
A_43_P11812	Biglycan (Bgn)	NM_017087	203010	114539	193016	1.69
A_42_P647599	Decorin (Dcn)	NM_024129	135143	139790	167452	1.20
A_43_P14911	Interleukin 1 beta (Il1b)	NM_031512	1009	398	452	1.13
A_43_P15252	Collagen, type II, alpha 1 (Col2a1)	NM_012929	102073	7983	8915	1.12
A_44_P536275	ADAM metallopeptidase with thrombospondin type 1 motif, 4 (Adamts4)	NM_023959	130	190	179	0.94
A_44_P508162	ADAM metallopeptidase with thrombospondin type 1 motif, 5 (Adamts5)	NM_198761	2672	2143	2049	0.95
A_44_P178519	ADAM metallopeptidase with thrombospondin type 1 motif, 7 (Adamts7)	NM_001047101	3743	6144	4944	0.80
A_44_P387222	Matrix metallopeptidase 1a (Mmp1a)	NM_001134530	23	148	160	1.08
A_44_P996729	Matrix metallopeptidase 2 (Mmp2)	NM_031054	114913	167698	163254	0.97
A_44_P318318	Matrix metallopeptidase 3 (Mmp3)	NM_133523	64292	213227	206101	0.96
A_44_P501112	Matrix metallopeptidase 9 (Mmp9)	NM_031055	785	45585	39302	0.86
A_44_P404861	Matrix metallopeptidase 10 (Mmp10)	NM_133514	35	4867	4695	0.96
A_44_P130333	Matrix metallopeptidase 11 (Mmp11)	NM_012980	222	65	70	1.08
A_44_P555271	Matrix metallopeptidase 12 (Mmp12)	NM_053963	5424	14439	18760	1.29
A_42_P606126	Matrix metallopeptidase 13 (Mmp13)	NM_133530	33573	185998	197176	1.06

The value of expression level in normal control, model control and APS-3c treatment group is the arithmetical average of normal control (or model control and APS-3c treatment) cRNA intensity in three chips and the ratio is the geometric average of three chips.
